# Management, control, and decision making in unexpected recurrent venous thromboembolism in COVID-19: a case report

**DOI:** 10.1186/s13256-023-03800-9

**Published:** 2023-03-19

**Authors:** Reza Zolfaghari Emameh, Jalal Heshmatnia

**Affiliations:** 1 Department of Energy and Environmental Biotechnology, National Institute of Genetic Engineering and Biotechnology (NIGEB), 14965/161, Tehran, Iran; 2 Chronic Respiratory Diseases Research Center (CRDRC), National Research Institute of Tuberculosis and Lung Diseases (NRITLD), Shahid Beheshti University of Medical Sciences, Tehran, Iran

**Keywords:** SARS-CoV-2, COVID-19, Deep-vein thrombosis, Vein thromboembolism, Case report, NOACs (new oral anticoagulants)

## Abstract

**Background:**

Coronavirus disease 2019 was spread worldwide, as a pandemic, from December 2019. Venous thromboembolism events can inflict patients with coronavirus disease 2019 during the hospitalization or convalescent period. Therefore, monitoring of these patients, in terms of venous thromboembolism events signs and symptoms, and timely management of antithrombotic agents are of great importance.

**Case report:**

A 45-year-old Iranian man, who is the first author of this case report, was infected by severe acute respiratory syndrome coronavirus 2 and displayed the typical signs and symptoms of coronavirus disease 2019. Although reverse transcription polymerase chain reaction for coronavirus disease 2019, and specific immunoglobulin M and immunoglobulin G against severe acute respiratory syndrome coronavirus 2, were negative at first, chest computed tomography scan showed the characteristic pattern of lung involvement of a coronavirus disease 2019 infection including bilateral and multilobar ground-glass opacities. At that time, there were no signs or symptoms of deep-vein thrombosis or pulmonary thromboembolism, so these were not investigated. About 30 hours after hospital discharge, the patient presented back to the hospital with acute-onset chest pain. We instantly tested his blood for D-dimer, and sent him to take a Doppler sonography of his lower legs and a chest computed tomography angiography in search of pulmonary thromboembolism and deep-vein thrombosis. Although we could confirm pulmonary thromboembolism with computed tomography angiography in our patient, there were no signs or symptoms of venous thromboembolism in his lower legs, and color Doppler sonography of lower limbs was normal. So, the patient was treated with rivaroxaban as an antithrombotic agent. After some days, he was discharged in good condition. About 1 month later, he was referred to our hospital because of left lower limb edema. Although he was under antithrombotic therapy, color Doppler sonography of lower limbs revealed acute deep-vein thrombosis of the left leg. Hence, we decided to shift antithrombotic therapy from rivaroxaban to warfarin, as it is more potent than rivaroxaban in recurrent venous thromboembolism and when taking new oral anticoagulants. Unlike rivaroxaban, which needs no blood test to monitor its efficacy but has a warning for signs and symptoms of bleeding, warfarin therapy must be monitored carefully by regular blood tests for prothrombin time and international normalized ratio to maintain them in the therapeutic range. The patient was informed about the bleeding cautions, and required regular check of prothrombin time and international normalized ratio to maintain them in the proper and advised range of treatment (international normalized ratio therapeutic range 2–3).

**Conclusion:**

In the case of unexpected recurrent venous thromboembolism in coronavirus disease 2019, especially when patients are taking rivaroxaban or other new oral anticoagulants, such drugs should be substituted by warfarin, with routine follow-up, to maintain the value of prothrombin time and international normalized ratio within the therapeutic range.

## Introduction

Severe acute respiratory syndrome coronavirus 2 (SARS-CoV-2), the causative agent of coronavirus disease 2019 (COVID-19), has spread worldwide since December 2019, so the World Health Organization (WHO) declared COVID-19 a pandemic disease around the world because of alarming spread, severity, and inaction [1–4]. The incubation period of COVID-19 is between 1 and 14 days post exposure to SARS-CoV-2; however, the median estimation for incubation period of COVID-19 was reported to be 5.1 days, with 95% confidence interval [5]. The clinical features of COVID-19 include dry cough, fatigue, fever (< 39 °C), chest discomfort, and sputum production, which is used for oral or nasopharyngeal swab sampling to perform reverse transcription polymerase chain reaction (RT-PCR) for molecular detection of SARS-CoV-2 [6]. The symptoms can develop into anorexia, dyspnea, myalgia, multiorgan dysfunction, hypoxia, respiratory failure, and death. Pulmonary thromboembolism (PTE) and deep-vein thrombosis (DVT) can easily complicate the natural history of disease from incubation to convalescent period, and thus must be treated by anticoagulant agents [7].

In this study, we report the development of recurrent venous thromboembolism (VTE) in a patient with COVID-19 after hospital discharge, despite proper prophylactic anticoagulant during the hospitalization period and therapeutic anticoagulant therapy after the first episode of PTE.

## Case report

**Fig. 1:**
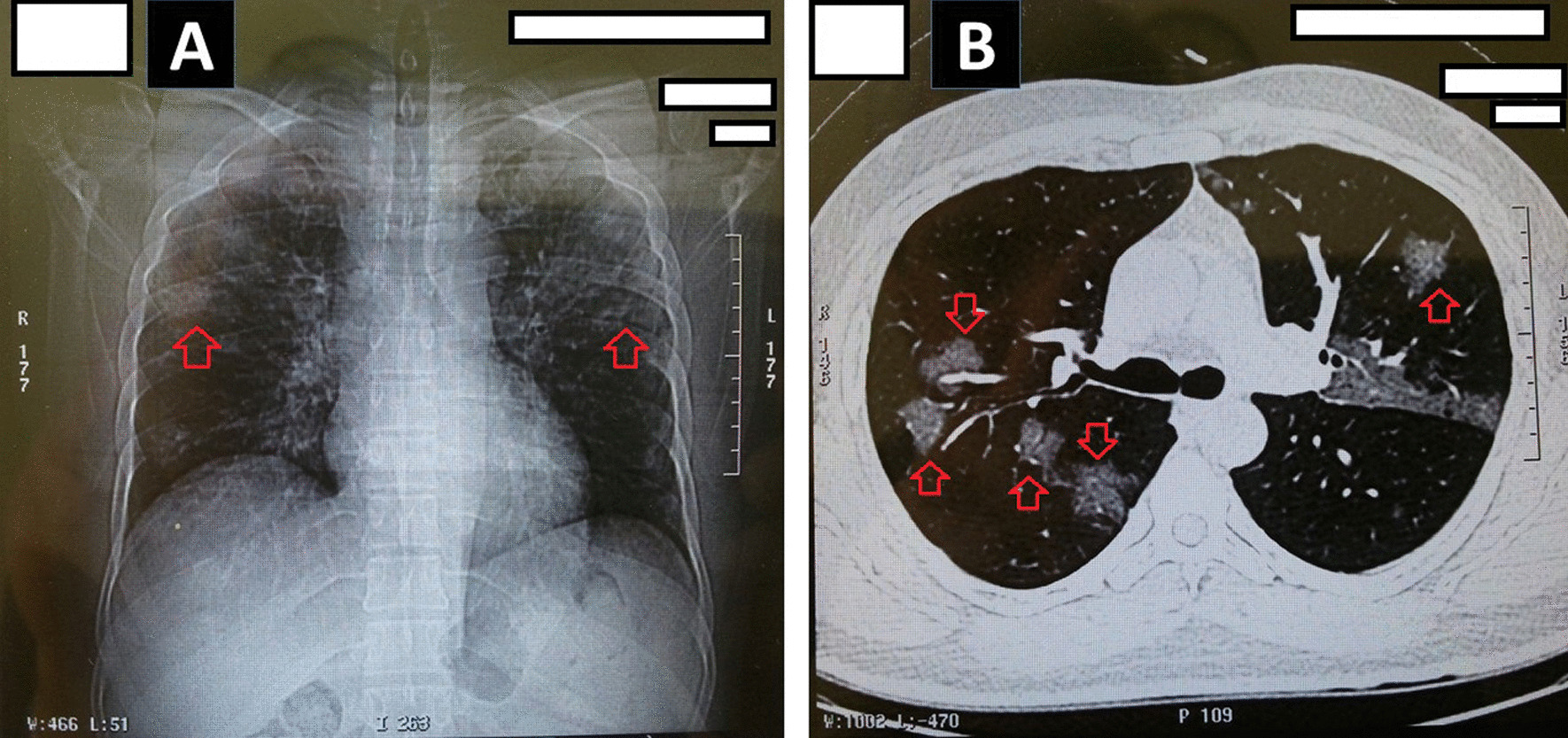
Chest computed tomography (CT) scan in the reported case with COVID-19. **A** Ground-glass opacification (GGO) in COVID-19 pneumonia; **B** bilateral and multilobar GGO in lungs. Arrows show the GGO in lungs

A 45-year-old Iranian man was infected by SARS-CoV-2 and displayed the general symptoms of COVID-19, including fever (< 38.5 °C), dry cough, fatigue, productive cough, blood oxygen saturation 88%, and chest pain. On arrival, a blood assay was performed to detect the COVID-19-specific antibody through enzyme-linked immunosorbent assay (ELISA). In the blood test, the anti-COVID-19 immunoglobulin M (anti-COVID-19 IgM) and anti-COVID-19 IgG were measured 0.18 μg/mL and 0.04 μg/mL, respectively (The reference value of anti-COVID 19 IgM and IgG were described as negative: < 0.9; borderline: 0.9–1.1 or positive: > 1.1). In addition, a sample was obtained by a sterile swab from nasopharynx for COVID-19 RT-PCR assay, of which the result was negative. A chest computed tomography scan (CT scan) was taken for detection of ground-glass opacification (GGO). The chest CT scan showed bilateral and multilobar GGO, specific characteristics of COVID-19 pneumonia (Fig. 1).

Since the GGO characteristics were observed following general symptoms, including fever (< 38.5 °C), dry cough, fatigue, pneumonia, and blood oxygen saturation 88%, during the COVID-19 pandemic, the patient was hospitalized as a typical case of COVID-19 pneumonia. Two weeks after hospital discharge, a second blood test was obtained to measure anti-COVID-19 IgM and anti-COVID-19 IgG by ELISA. In this analysis, anti-COVID-19 IgM and anti-COVID-19 IgG were 3.80 μg/mL and 20.40 μg/mL, respectively (the reference values of IgM and Ig G anti-COVID 19 were described as negative < 0.9, borderline 0.9–1.1, or positive > 1.1). No signs or symptoms of VTE including DVT and PTE were observed during the course of hospitalization, so Doppler sonography of the lower limbs and CT angiography of chest were not taken.

**Fig. 2:**
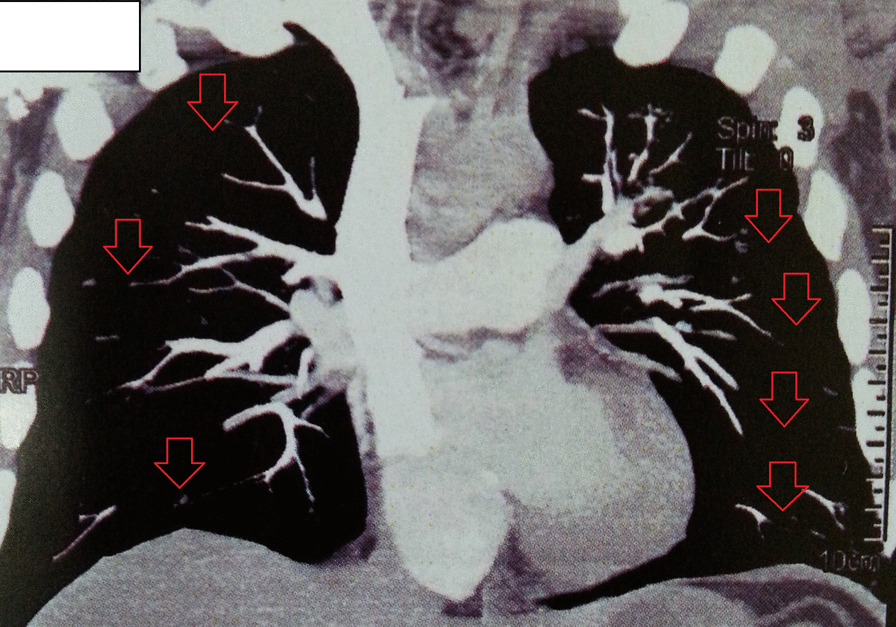
CT angiography (CTA) in the reported case with COVID-19. The figure shows acute pulmonary thromboembolism (PTE), supported by filling defects in lateral segment branch of right lower lobe (RLL) and posteromedial segments of left lower lobe (LLL). Arrows show the filling defects due to PTE

The onset of chest pain and tachycardia about 30 hours post discharge from hospital urged us to measure D-dimer in the blood test. The value of D-dimer was > 7.99 μg/mL (reference value 0.0–0.50 μg/mL). Although Doppler sonography of lower limbs revealed no signs of acute DVT, a CT angiography was performed, which depicted the filling defects in lateral segment branch of right lower lobe (RLL) and posteromedial segments of left lower lobe (LLL) pulmonary arteries, in favor of acute pulmonary thromboembolism (PTE) (Fig. 2).

Thereafter, 15 mg rivaroxaban tablets were prescribed twice a day for 21 days, which was followed by 20 mg rivaroxaban tablets once a day for the rest of treatment period. After about 1 month, while the patient was on therapeutic anticoagulant agent, an edema was noticed around the left ankle supporting the diagnosis of DVT. Sonography was performed on the lower limb veins. Our findings revealed increased diameter in popliteal vein (POPV) of the left foot, with echogenic materials in it and noncompressible characteristics of the veins, which were in favor of acute DVT diagnosis. To manage and control recurrent VTE, the administration of rivaroxaban was stopped and anticoagulant therapy was focused on using warfarin tablets, because warfarin is more potent than new oral anticoagulants (NOACs) when there is a recurrent VTE, despite using NOACs, or a specific condition such as antiphospholipid antibody syndrome. Initially, one 5 mg warfarin tablet per day was prescribed for 1 week, simultaneously while administering Clexane ampule 6000 units every 12 hours until the international normalized ratio (INR) reached a therapeutic level for two consecutive days to prevent reverse thrombotic side effects of warfarin, and then the warfarin dose was adjusted according to the prothrombin time (PT) and INR values (between 2 and 3) in the blood.

## Discussion

Our serology study detected a high concentration of anti-COVID-19 IgM and anti-COVID 19 IgG after 2 weeks of general symptom onset. In addition, the blood concentration level of anti-COVID-19 IgG was higher than that of anti-COVID-19 IgM. The increase in IgM and IgG antibodies against the coronavirus confirmed the COVID-19 pneumonia in our patient. Our results were compatible with the outputs of another study, performed by Shu *et al*. They reported that the anti-COVID-19 IgM and anti-COVID-19 IgG blood concentration levels peaked about 18 and 23 days post symptom onset, and then anti-COVID-19 IgM decreased to baseline level at about day 36, whereas anti-COVID-19 IgG remained at a high blood concentration level for a quite long period [8]. COVID-19 pneumonia was also indicated by the GGO characteristic images obtained by chest CT scan, even though RT-PCR result was negative, which can be consistent with higher and lower sensitivity of CT scan and RT-PCR in detection of COVID-19, respectively. The previous studies defined chest CT scan and RT-PCR as having a sensitivity of 97% and 59.8%, respectively, in COVID-19 detection [9, 10]. Some studies demonstrated that both high blood concentration level of D-dimer and tachycardia are associated with severity of COVID-19, in the absence of an established PTE or DVT [11, 12]. As COVID-19 infection is a potent risk factor for VTE events, it is wise to use a prophylactic dose of anticoagulants during admission. Despite using prophylactic and therapeutic doses of antithrombotic agents, in some cases PTE or DVT can occur because of a hypercoagulable state, which is produced by severe inflammation in this infection [13, 14]. In our case presentation, recurrent PTE or DVT occurred despite using the standard dose of rivaroxaban as an anticoagulant agent during the second admission because of acute PTE. In this case, a shifting approach was performed to substitute rivaroxaban with warfarin to manage and control the recurrent VTE. Within this context, it is critical to tightly control the levelsof PT and INR, which must be measured frequently and maintained in the therapeutic range of 2–3 by adjusting the dose of warfarin proportionately [15]. The patient was notified that it is necessary to check the INR level whenever taking new medication for any reason, because every medication can disturb the therapeutic range of INR and pose a threat of bleeding or new VTE on our patient.

In our case we have to preclude other conditions that may predispose the patient to recurrent VTE or PTE such as occult malignancies and rheumatologic disorders, for example, anti-phospholipid antibody and inherited thrombophilia—including protein S and C deficiency, factor V Leiden, prothrombin G20210A mutation, and anti-thrombin deficiency. This patient underwent a dedicated surveillance for mentioned predisposing factors, and we found no evidence for such condition on 2-year follow-up.

## Conclusion

COVID-19 increases the risk of VTE event onset in hospitalized and nonhospitalized patients. Therefore, the administration of a prophylactic and therapeutic dose of anticoagulant agents, such as rivaroxaban or warfarin, has great value in controlling VTE occurrence during the hospitalization and convalescent period.

The most important point of our case is the recurrent VTE events, despite a proper prophylactic and therapeutic drug regimen against VTE during acute and chronic phase of COVID infection. Although there was a delay between COVID-19 infection and VTE onset presentations, this case has great value for clinicians, including general practitioners or internal medicine specialists, who must be more careful and suspicious of possible VTE events among patients with COVID-19, despite applying dedicated therapeutic strategies against VTE. During 2 years of follow-up, fortunately no sign or symptoms of aforementioned predisposing conditions to VTE events have been found in our patient, which seems to be a very good indication of COVID-19 infection as a culprit for recurrent VTE events.

## Data Availability

The data supporting this study has been included within the manuscript by the author.

## Abbreviations

COVID-19: Coronavirus disease 2019
CTA: Computed tomography angiography
CT scan: Computed tomography scan
DVT: Deep-vein thrombosis
ELISA: Enzyme-linked immunosorbent assay
EMG: Electromyography
GGO: Ground-glass opacification
INR: International normalized ratio
PT: Prothrombin time
PTE: Pulmonary thromboembolism
RLL: Right lower lobe 193
SARS-CoV-2: Severe acute respiratory syndrome coronavirus 2
VRT: Volume rendering technique
VTE: Venous thromboembolism
NOACs: New oral anticoagulants
